# Chitosan can improve antimicrobial treatment independently of bacterial lifestyle, biofilm biomass intensity and antibiotic resistance pattern in non-aureus staphylococci (NAS) isolated from bovine clinical mastitis

**DOI:** 10.3389/fmicb.2023.1167693

**Published:** 2023-04-21

**Authors:** Maria Laura Breser, Lucia Tisera, Maria Soledad Orellano, Luciana Paola Bohl, Paula Isaac, Ismael Bianco, Carina Porporatto

**Affiliations:** ^1^Instituto Multidisciplinario de Investigación y Transferencia Agroalimentaria y Biotecnológica (IMITAB-CONICET), Universidad Nacional de Villa María, Villa María, Argentina; ^2^Instituto Académico Pedagógico de Ciencias Básicas y Aplicadas, Universidad Nacional de Villa María, Villa María, Argentina; ^3^University of the Basque Country UPV/EHU. Responsive Polymer Therapeutics Group (POLYMAT), San Sebastián, Spain; ^4^Centro de Excelencia en Productos y Procesos de Córdoba (CEPROCOR), Ministerio de Industria, Comercio, Minería y Desarrollo Científico Tecnológico, Córdoba, Argentina

**Keywords:** biofilm, antibiotic resistance, chitosan, cloxacillin, biofilm and antibiotic resistance association, non-aureus staphylococcus

## Abstract

Bovine mastitis is the most frequent and costly disease that affects dairy cattle. Non-aureus staphylococci (NAS) are currently one of the main pathogens associated with difficult-to-treat intramammary infections. Biofilm is an important virulence factor that can protect bacteria against antimicrobial treatment and prevent their recognition by the host’s immune system. Previously, we found that chronic mastitis isolates which were refractory to antibiotic therapy developed strong biofilm biomass. Now, we evaluated the influence of biofilm biomass intensity on the antibiotic resistance pattern in strong and weak biofilm-forming NAS isolates from clinical mastitis. We also assessed the effect of cloxacillin (Clx) and chitosan (Ch), either alone or in combination, on NAS isolates with different lifestyles and abilities to form biofilm. The antibiotic resistance pattern was not the same in strong and weak biofilm producers, and there was a significant association (*p* ≤ 0.01) between biofilm biomass intensity and antibiotic resistance. Bacterial viability assays showed that a similar antibiotic concentration was effective at killing both groups when they grew planktonically. In contrast, within biofilm the concentrations needed to eliminate strong producers were 16 to 128 times those needed for weak producers, and more than 1,000 times those required for planktonic cultures. Moreover, Ch alone or combined with Clx had significant antimicrobial activity, and represented an improvement over the activity of the antibiotic on its own, independently of the bacterial lifestyle, the biofilm biomass intensity or the antibiotic resistance pattern. In conclusion, the degree of protection conferred by biofilm against antibiotics appears to be associated with the intensity of its biomass, but treatment with Ch might be able to help counteract it. These findings suggest that bacterial biomass should be considered when designing new antimicrobial therapies aimed at reducing antibiotic concentrations while improving cure rates.

## 1. Introduction

Bovine mastitis (BM) is the most prevalent disease that affects dairy cattle. It compromises the health of dairy herds and leads to serious economic losses that can impact the entire production chain (Hogeveen et al., 2011; Aghamohammadi et al., 2018; Heikkilä et al., 2018). The costs are related to the intervention, treatment, and monitoring of infection, and are compounded by a significant reduction in the quality and quantity of the milk produced (Vissio et al., 2015; Aghamohammadi et al., 2018; Heikkilä et al., 2018). Coagulase-negative Staphylococcus, recently named non-aureus staphylococci (NAS), are one of the most prevalent pathogens associated with BM (De Visscher et al., 2016; Condas et al., 2017; Taponen et al., 2017). They are associated with persistent intramammary infection (IMI), decreased milk production (Heikkilä et al., 2018), and elevated somatic cell counts (SCC; Supré et al., 2011; Fry et al., 2014). In the absence of effective immunoprevention therapies, antibiotics have become the main means to deal with this pathology. However, their indiscriminate use and the high mutation rates of pathogens have led to the emergence of multiresistant bacteria, against which antibiotics are increasingly less effective (Chantziaras et al., 2014; Lhermie et al., 2017; De Jong et al., 2018; Kim et al., 2019; Van Boeckel et al., 2019). In fact, NAS are currently the most resistant mastitis-causing pathogens (de Oliveira et al., 2016; Dorneles et al., 2019; Kim et al., 2019), since they have higher mutation rates and are more capable of horizontal gene transfer than other species. This means they might act as reservoirs and help spread different virulence factors (Bal et al., 2010; Sampimon et al., 2011; Taponen et al., 2017).The highest cure rates for the clinical and subclinical forms of the disease are usually obtained when antibiotics are applied during dry-off (Rabiee and Lean, 2013). Nevertheless, only 66% of the udders infected before this period are clinically cured afterwards (Lipkens et al., 2019).

Bacterial biofilms may lessen exposure to antibiotics and offer general protection against adverse environmental factors, so they may further hamper the efficacy of antimicrobial therapies (Lebeaux et al., 2014; Tremblay et al., 2014; de Oliveira et al., 2016; Srednik et al., 2017; Breser et al., 2018). Biofilms not only prevent antibiotics from acting directly on bacterial growth, but can also allow the entry of low or suboptimal concentrations of these compounds into their structure (Flemming et al., 2016; Ster et al., 2017). Such concentrations may exert selection pressure and favor the development of resistance in the bacteria within the biofilm (Flemming et al., 2016). Moreover, strong biofilm-forming strains have been described to cause more severe tissue damage than weak producers. More than 85% of the NAS isolated from BM can reportedly grow in biofilms (Tremblay et al., 2014; Felipe et al., 2017; Srednik et al., 2017). Previously, we found that NAS isolates from chronic BM which were refractory to different antibiotic protocols were able to develop strong biofilm biomass (Breser et al., 2018). This indicates that biofilm growth could play an important role in IMI chronicity.

On the other hand, chitosan (Ch) is a natural, biocompatible, and biodegradable polymer with many well-documented biological applications. Its reported antimicrobial activity against a wide range of microorganisms is mainly attributed to its cationic nature (Muxika et al., 2017; Verlee et al., 2017). When we tested it on its own or combined with antibiotics, it had remarkable antimicrobial effects on *Staphylococcus* isolates from clinical and chronic IMIs, whether in their planktonic form, in preformed biofilms, or in intracellular infections (Breser et al., 2018; Felipe et al., 2019).

Given the continuous increase in antimicrobial resistance among mastitis pathogens and the associated reduction in clinical and bacteriological cure rates, chitosan and other similar materials could be useful for the design of new control strategies. These strategies should consider the role of well-established biofilms in the emergence of resistance, which so far remains unclear. Accordingly, the present study evaluated the influence of biofilm forming strength on the patterns of antibiotic resistance in NAS isolates from clinical mastitis. It also assessed the effectiveness of combining Ch with an antibiotic against different bacterial lifestyles.

## 2. Materials and methods

### 2.1. Bacterial isolate classification, growth conditions, and reagents

A total of 110 non-aureus staphylococci (NAS) isolates from clinical BM were collected at 14 independent local dairy farms. NAS were isolated from milk samples from quarters with clinical signs, a positive Californian Mastitis Test (CMT), and SCC ≥ 250.000 cells/ml. The samples were cultured at 37°C for 24 h on Trypticase soy agar (TSA) plates containing 5% sheep blood. All the experiments were carried out under the supervision and with the approval of the Institutional Ethics Committee at the National University of Villa María (UNVM) which monitors experiments with animals, as well as in accordance with international guidelines for the use and handling of pathogenic microorganism isolates from mastitis (Hogan et al., 1999). The identity of the isolates had been confirmed earlier by amplification and partial sequencing of the 16S rRNA gene and MALDI-TOF, and they were classified taxonomically as NAS species (Felipe et al., 2017).

To categorize the intensity of their biofilms, a biofilm formation assay was performed on an abiotic surface and the biomass produced was quantified as previously described (Breser et al., 2018). Briefly, 100 μl of bacterial suspensions (1 × 10^7^ CFU/ml) in Trypticase soy broth (TSB) were added into individual wells on flat polystyrene microtiter plates. The plates were statically incubated for 24 h at 37°C, to allow the cells to bind and biofilm to form. The supernatants were discarded and the biofilms were washed twice with sterile PBS to remove non-adherent bacteria, air dried, and stained with a crystal violet (CV) solution (0.5% w/v) for 5 min. The excess dye was washed and the plates were left to dry for 24 h. The dye bound to each well was resuspended in 200 μl of 96% alcohol. After 20 min of incubation at room temperature, 100 μl were taken from each well and transferred into a new 96-well plate. The absorbance of the eluates was measured at 590 nm with a Multiskan GO microplate spectrophotometer reader (TermoFisher Scientifc), and expressed as optical density (OD) values. The OD values were used to assess the degree of biofilm adhesion to the contact surface. The negative control (NC) consisted of wells cultured with TSB. The OD_NC_ value was obtained by considering three standard deviations above the mean value of the negative control. An isolate was considered a weak biofilm producer (WBP) when OD_NC_ < OD of the isolate ≤4 OD_NC_. On the other hand, when the OD of the isolate >8 OD_NC_ (OD_590nm_ between 2 and 3.15)_,_ it was classified as a strong biofilm producer (SBP; Li et al., 2012). In all the assays, *S. epidermidis* strain ATCC 12288 was used as a negative control for biofilm formation (Zhang et al., 2003). Two different groups of 10 NAS isolates each were selected from the collection on the basis of their biofilm biomass intensity (strong vs. weak). To maintain homogeneous species distribution, each group included two isolates of each of the most frequently found species: *S. chromogenes*, *S. simulans*, *S. xylosus*, *S. epidermidis*, and *S. haemolyticus*.

As a quality control for the microbiological assays, the following *Staphylococcus aureus* (*S. aureus*) strains were used: ATCC 29213, ATCC 25923, methicillin-resistant (MRSA) ATCC 43300, and ATCC 35984. The isolates and the reference strains were stored at −80°C in a nutrient broth containing 20% glycerol. The inocula were prepared in TSB at 37°C, 18 to 24 h before carrying out the assays. They were adjusted with DensiCHEK Plus (bioMérieux SA, Marcy-l’Étoile, France) according to the McFarland scale, and the values were corroborated by plate counting.

Low molecular weight chitosan (Ch; 50–90 kDa powder with ≥85% deacetylation), cloxacillin (Clx) powder, and crystal violet (CV) were purchased from Sigma-Aldrich (St Louis, MO, USA). The antibiotic disks and the nutrient media were purchased from Britania (CABA, BA, Argentina).

### 2.2. Susceptibility test

The antimicrobial susceptibility of the isolates was determined by the standard disk diffusion method, following the guidelines by the guidelines of Clinical and Laboratory Standards Institute (CLSI) (2020). The isolates were recovered on fresh TSA 18–24 h prior to the test. The direct colonies were suspended and adjusted at 0.5 on the McFarland scale. The suspensions were swabbed across Mueller-Hilton agar plates; antibiotic disks were placed on the surface, and the plates were incubated for 18–20 h at 35°C. Afterwards, the zone of inhibition around the disks was measured with a calibrated ruler and interpreted according to CLSI breakpoints. The disks used were penicillin (PEN) 10 Units, ampicillin (AMP) 10 μg, cefoxitin (FOX) 30 μg, erythromycin (ERY) 15 μg, and rifampicin (RIF) 5 μg. A cefoxitin disk was used as an indicator of methicillin susceptibility.

### 2.3. Determination of MIC and MBC

The minimum inhibitory concentrations (MIC) of Clx and Ch were determined through a broth microdilution assay according to the CLSI guidelines [Clinical and Laboratory Standards Institute (CLSI), 2020]. Bacterial suspensions (1×10^5^ CFU/mL) were cultured for 24 h at 37°C in 96-well bottom-plates (Deltalab, Barcelona, Spain), with different concentrations of Clx (0.025 to 16 μg/ml), Ch (200 μg/ml), or combinations of both. The planktonic minimum bactericidal concentration (P-MBC) was found by seeding the bacteria on TSA plates with concentrations of the antimicrobial compounds which were equal to or greater than the MIC, and assessing viability after 24 h (Breser et al., 2018).

### 2.4. Bacterial viability in preformed biofilms

The viability of bacteria within biofilms was measured after different treatments as previously described (Breser et al., 2018). Briefly, 100 μl of bacterial suspensions (1×10^7^ CFU/mL) were placed into 96-well flat-plates (Deltalab), and a final volume of 200 μl in each well was obtained by adding TSB. The plates were statically incubated for 24 h at 37°C, to allow the cells to bind and biofilms to form. After that, the supernatants were discarded and the biofilms were washed twice with sterile PBS to remove non-adherent bacteria. These preformed biofilms were treated for 24 h at 37°C with different concentrations of Clx (64 to 2048 μg/mL), Ch (200 μg/ml), or combinations of both. The biofilm minimum bactericidal concentration (B-MBC) was determined by measuring bacterial viability within the biofilms biomass, after it was disaggregated and seeded on TSA plates.

### 2.5. Viability analysis by flow cytometry

Bacterial suspensions were obtained from planktonic cultures grown for 24 h at 37°C in TSB (control), or with the addition of different concentrations of Clx (4 to 0.065 μg/ml), Ch (200 μg/ml), or combinations of both. Homogeneous suspensions were also prepared with the preformed biofilms, which were disaggregated and filtered after growing for 24 h at 37°C in TSB (control), or in the presence of Clx (8 to 2048 μg/ml), Ch (200 μg/ml), or combinations of both. Bacterial viability was evaluated in both kinds of suspension with a LIVE/DEAD BacLight Bacterial Viability Kit (ThermoFisher Scientific, CA, USA), according to the manufacturer’s instructions. The suspensions were analyzed with an ACCURI C6 cytometer (BD Bioscience, CA, USA), and the data were processed on FlowJo software (Tree Star, OR, USA; Breser et al., 2018).

### 2.6. Statistical analysis

The parametric data were statistically analyzed by one or two-way ANOVA, with a Bonferroni *post hoc* test, while non-parametric data were statistically analyzed with Kruskal Wallis one-way analysis of variance. The number of independent replicates in each assay has been specified in the corresponding figures. To control the variability of the isolates, some analyses were conducted with a two-factor factorial design, in which the first factor was the treatment and the second factor was the isolate. R software was used to process the information (R Core Team, 2020), and the graphs were made on GraphPad Prism 5.0 (GraphPad Software, Inc., CA, USA).

## 3. Results

### 3.1. Antimicrobial resistance pattern in strong and weak biofilm-producers NAS isolates

**Figure 1:**
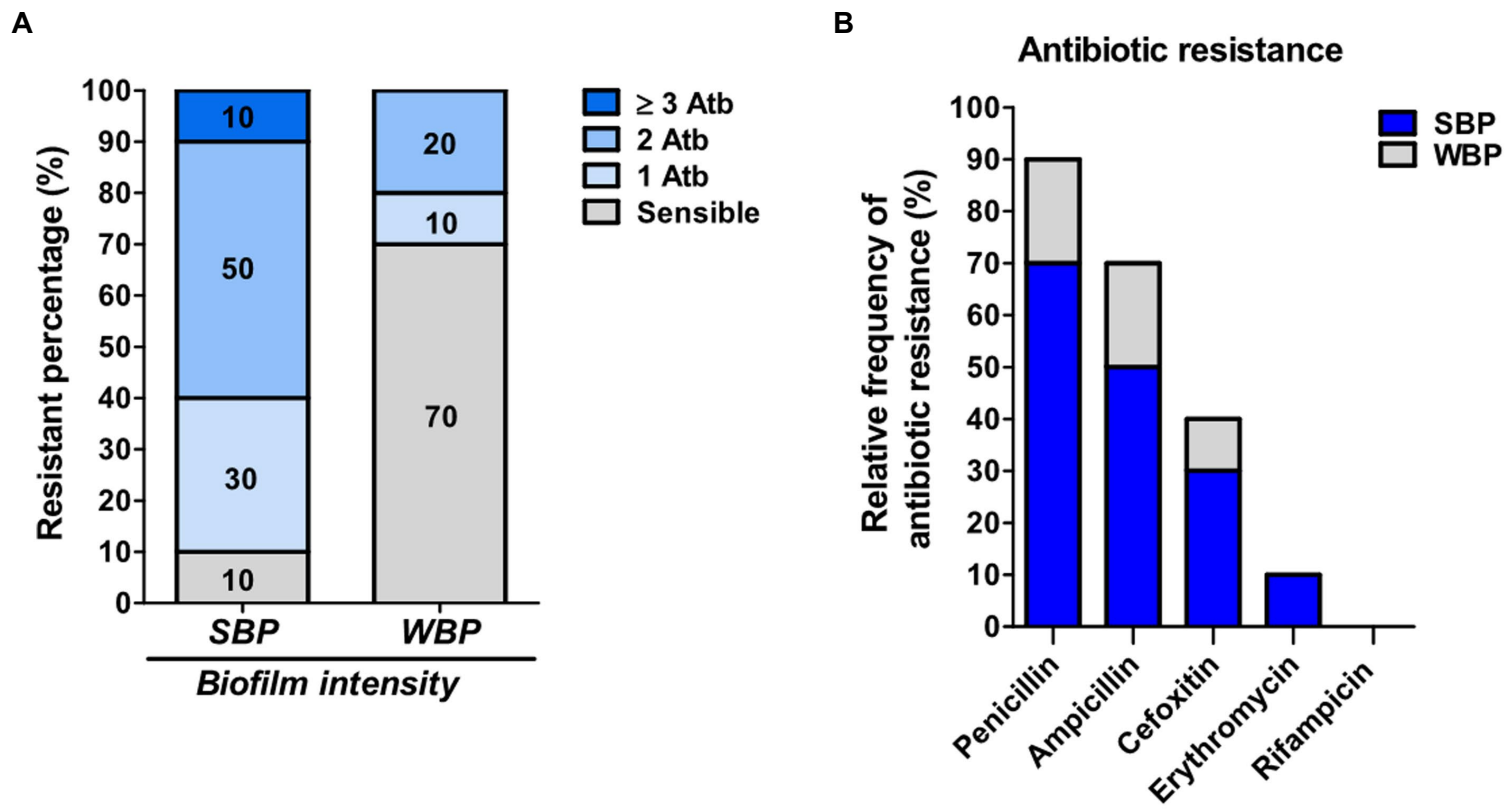
Antibiotic resistance in SBP and WBP NAS isolates from clinical bovine mastitis. **(A)** Percentage of resistance to antibiotics in SBP and WBP NAS isolates. **(B)** Relative frequency of resistance to penicillin, ampicillin, cefoxitin, erythromycin and rifampicinin SBP and WBP NAS isolates.

Even though biofilm formation can be an important virulence factor, not enough is known about its direct influence on antibiotic resistance. We evaluated the antimicrobial resistance pattern in NAS isolates in relation to their biofilm-forming ability. The isolates were selected from a collection and divided into two groups of 10 each, depending on whether they were strong biofilm producers (SBPs) or weak biofilm producers (WBPs; Felipe et al., 2017; Breser et al., 2018). The first step was to compare the extracellular components in mature SBP and WBP biofilms (Oniciuc et al., 2016). In both cases, over 88% of the biofilm was removed after treatment with sodium metaperiodate (NaIO4), which degrades exopolysaccharides, and about 20% was removed after treatment with Proteinase K, which degrades proteins. This means that the composition of the biofilm matrix was similar for the two groups (Supplementary Figure). We then assessed the susceptibility of the isolates to the main antibiotic families used to control mastitis, and found that SBPs and WBPs had different antimicrobial resistance patterns (Tables 1A,B; Figure 1). According to the antibiogram, 70, 50, 30, and 10% of the SBPs were, respectively, resistant to penicillin, ampicillin, cefoxitin, and erythromycin (Table 1A), while the percentages were 20, 20, 10, and 0% for WBPs (Table 1B). All the isolates were sensitive to rifampicin. Only 10% of the SBPs were sensitive to different antibiotic families; in the case of WBPs it was 70% (Figure 1). The relative frequency of resistance was 0.9 for SBPs; i.e., 9 to 10 of these isolates were resistant to one or more antibiotics. This value was 0.3 (3 to 10 isolates) for WBPs (Figure 1; Table 1). An odd-ratio analysis showed a significant association (*p* ≤ 0.01) between biofilm biomass intensity and antibiotic resistance; and an individual association between biofilms intensity and penicillin resistance (*p* ≤ 0.01).

**Table 1:** Antibiotic susceptibility in SBP and WBP NAS isolates from clinical bovine mastitis.

Isolates	PEN	AMP	FOX	ERY	RIF	Resistance(#)
A. Strong biofilms producer NAS						
*SBP1*	R	R	R	S	S	3
SBP2	R	S	R	S	S	2
SBP3	R	R	S	S	S	2
SBP4	R	S	S	S	S	1
SBP5	R	R	S	S	S	2
SBP6	S	S	S	R	S	1
SBP7	S	S	S	S	S	0
SBP8	S	S	R	S	S	1
SPB9	R	R	S	S	S	2
SBP10	R	R	S	S	S	2
Resistance (%)	70	50	30	10	0	
B. Weak biofilm producer NAS						
WBP1	S	S	S	S	S	0
WBP2	S	S	S	S	S	0
WBP3	S	S	S	S	S	0
WBP4	S	S	S	S	S	0
WBP5	S	S	S	S	S	0
WBP6	I	I	S	S	S	0
WBP7	R	R	S	S	S	2
WBP8	S	S	S	S	S	0
WBP9	R	S	R	S	S	2
WBP10	S	R	S	S	S	1
Resistance (%)	20	20	10	0	0	

R, resistant; S, susceptible. AMP, ampicillin; PEN, penicillin G; FOX, cefoxitin; ERY, erythromycin; and RYF, rifampicin. The number of antibiotics to which each NAS isolate was resistant is shown in the last column.The percentage of resistance to each antibiotic is shown in the last row. A. Strong biofilms producer NAS isolates. B. Weak biofilms producer NAS isolates.

### 3.2. Effect of cloxacillin on planktonic cultures and preformed biofilms

**Figure 2:**
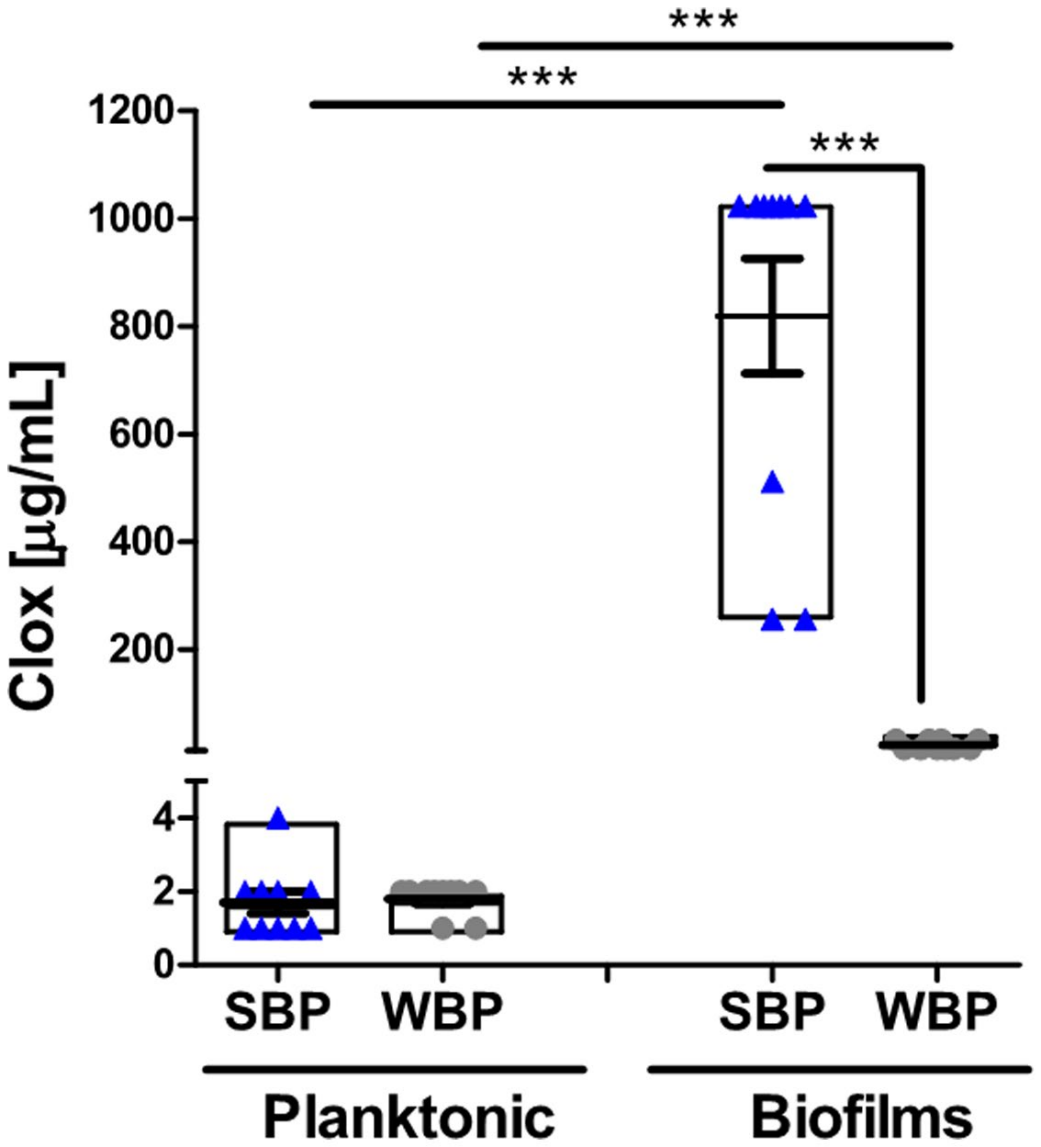
Minimum bactericidal concentration of cloxacillin for SBP and WBP NAS isolates in planktonic cultures and preformed biofilms. The bar graph shows the cloxacillin MBC in planktonic cultures and biofilms measured by a plate count assay. Data are shown as the mean of each isolate ± SEM and box plot distribution. These experiments were performed three independent times with three biological replicates of each of the 10 SBP and WBP isolates. The *p* values *** < 0.001 were obtained with a Kruskal-Wallis test.

The minimum bactericidal concentration was determined by measuring the viability of SBPs and WBPs in planktonic cultures (P-MBC) and in preformed biofilms (B-MBC). The cloxacillin (Clx) concentration needed to kill bacteria in the biofilms (256 to 1,024 μg/ml for SBPs; 16 to 32 μg/ml for WBPs) was significantly higher (*p* ≤ 0.001, Kruskal-Wallis test) than for their planktonic counterparts (1 to 4 μg/ml for SBPs and WBPs; Table 2; Figure 2). In fact, the concentration required to kill SBPs in preformed biofilms was 128 to 1,024 times the one needed when they grew planktonically. For WBPs, the B-MBC was 8–16 times the P-MBC. The differences between the two groups were directly associated with their biofilm-forming ability (*p* ≤ 0.001, Kruskal-Wallis test; Table 2; Figure 2).

**Table 2:** Susceptibility of SBP and WBP NAS isolates to cloxacillin in planktonic cultures and preformed biofilms.

	Planktonic cultures		
Isolates	P-MBC [μg/ml]	B-MBC [μg/ml]	B-MBC/P-MBC
A. Strong biofilms producer NAS			
SBP1	1	1,024	1,024
SBP2	2	1,024	512
SBP3	2	1,024	512
SBP4	2	1,024	512
*SBP5*	1	1,024	1,024
SBP6	2	1,024	512
SBP7	4	512	128
SBP8	1	1,024	1,024
SBP9	1	256	256
SBP10	1	256	256
B. Weak biofilms producer NAS			
WBP1	2	32	16
WBP2	1	16	16
WBP3	2	16	8
WBP4	2	32	16
WBP5	2	16	8
WBP6	1	16	16
WBP7	2	32	16
WBP8	2	16	8
WBP9	2	32	16
WBP10	2	16	8

A. Strong biofilms producer bacteria. B. Weak biofilms producer bacteria. Minimum bactericidal concentration of cloxacillin in planktonic cultures (P-MBC), and in preformed biofilms (B-MBC). Bactericidal ratio between preformed biofilms and planktonic cultures (B-MBC/P-MBC). The breakpoint for the minimum inhibitory concentration (MIC) of cloxacillin was taken from the CLSI guidelines for penicillinase-stable penicillins in NAS (≥0.5 μg/ml).

**Figure 3:**
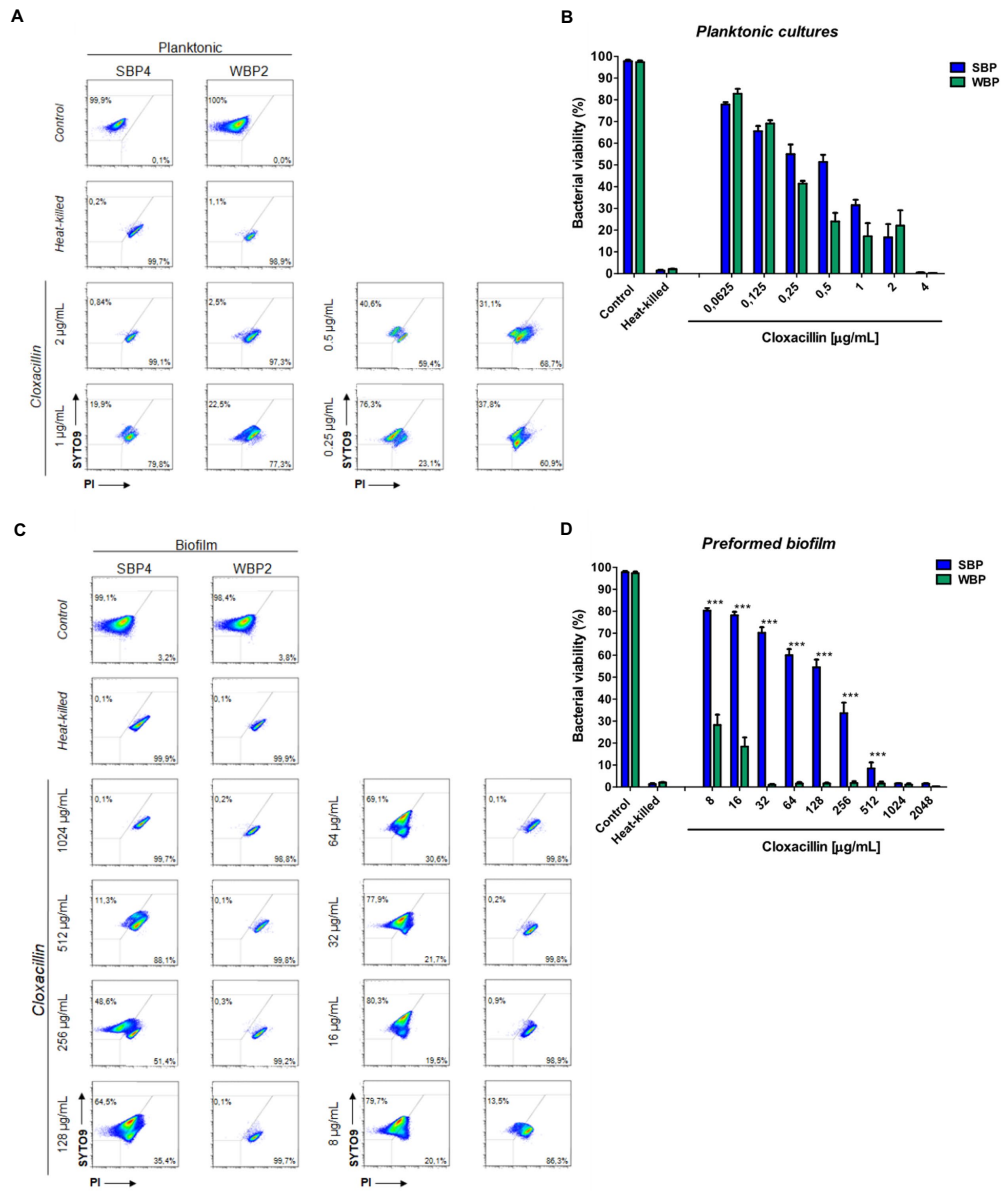
Bacterial viability of SBP and WBP NAS isolates in planktonic cultures and preformed biofilms after treatment with cloxacillin. **(A)** Bacterial viability of SBPs and WBPs in planktonic cultures grown in TSB or treated with different concentrations of cloxacillin, analyzed by flow cytometry using SYTO9 and PI dyes. **(B)** Bacterial viability percentages of SBPs and WBPs in planktonic cultures. **(C)** Bacterial viability of SBPs and WBPs in preformed biofilms grown in TSB or treated with different concentrations of cloxacillin, analyzed by flow cytometry using SYTO9 and PI dyes. **(D)** Bacterial viability percentages of SBPs and WBPs in preformed biofilms. These experiments were performed four independent times with three biological replicates of each of the 10 SBP and WBP isolates. Data were analyzed with one-way ANOVA followed by Bonferroni post-hoc, and are shown as mean ± SEM. The *p* values * < 0.05, ** < 0.01, and *** < 0.001 were considered significant.

A flow cytometry analysis with SYTO9 and PI dyes was performed to assess bacterial viability in terms of membrane integrity, after SBPs and WBPs in planktonic cultures and preformed biofilms were incubated with different Clx concentrations. An isolate from each group was selected to build the density plots (Figures 3A,C), and bar graphs show the percentages of bacterial viability for all the isolates in each group (Figures 3B,D). After exposure to different concentrations of the antibiotic, bacterial viability was similar for both groups in the planktonic cultures (Figures 3A,B). In contrast, viability was significantly higher for SBPs than for WBPs when they were treated with the same Clx concentration in the preformed biofilms (Figures 3C,D). Interestingly, SBPs appeared to be generally much more protected than WBPs inside the biofilms, regardless of their specific antibiotic resistance pattern (Figures 3C,D). The flow cytometry data confirm that the antibiotic concentrations needed to kill bacteria in preformed biofilms may be closely related to biofilm-forming ability (Figures 2, 3).

### 3.3. Chitosan and cloxacillin reduce the protection conferred by biofilm

**Figure 4:**
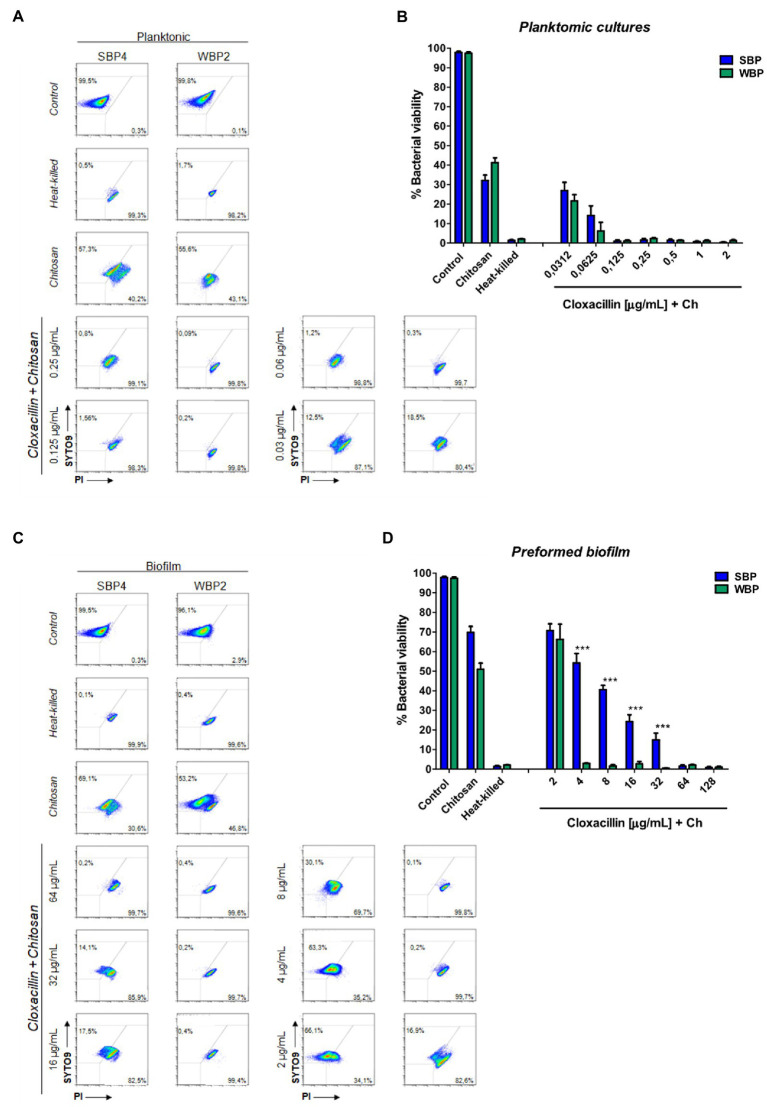
Minimum bactericidal concentration of combined chitosan and cloxacillin in SBP and WBP NAS isolates in planktonic cultures and preformed biofilms. **(A)** Bacterial viability of SBPs and WBPs in planktonic cultures grown in TSB or treated with different concentrations of cloxacillin and chitosan, analyzed by flow cytometry using SYTO9 and PI dyes. **(B)** Bacterial viability percentages of SBPs and WBPs in planktonic cultures. **(C)** Bacterial viability of SBPs and WBPs in preformed biofilms grown in TSB or treated with different concentrations of cloxacillin and chitosan, analyzed by flow cytometry using SYTO9 and PI dyes. **(D)** Bacterial viability percentages of SBPs and WBPs in preformed biofilms. These experiments were performed four independent times with three biological replicates of each of the 10 SBP and WBP isolates. Data were analyzed with one-way ANOVA followed by Bonferroni post-hoc, and are shown as mean ± SEM. The *p* values * < 0.05, ** < 0.01, and *** < 0.001 were considered significant.

Biofilms are known to protect bacteria against antimicrobial therapies, but more information is needed about the influence of biomass intensity on the degree of protection conferred. Earlier, we observed that combining Clx with Ch made it possible to reduce the concentration of the antibiotic required to kill NAS isolates within biofilms (Breser et al., 2018). Here, we used plate counting and flow cytometry to explore the effects of Clx and Ch (alone or combined) on NAS isolates with different biofilm-forming abilities. As before, the bacteria were evaluated in planktonic cultures and preformed biofilms. The combination of Clx and Ch enabled a significant reduction in the Clx concentration needed to kill both SBPs and WBPs, in planktonic cultures and in preformed biofilms (Table 3; Figure 4). In planktonic cultures, the antibiotic concentrations needed to kill SBPs and WBPs were, respectively, 8–32 times and 16–32 times those required to achieve the same purpose in combination with Ch (Table 3A). In the preformed biofilms, the combination of Clx and Ch resulted in a 4-to 16-fold reduction in the amount of the antibiotic needed to kill SBPs. For WBPs, this reduction was 4-to 8-fold (Table 3B; Figure 4). Furthermore, Ch had strong antimicrobial activity on its own, regardless of bacterial growth or the intensity of the biofilm (Figures 4). It reduced the viability of SBPs by 67% in planktonic cultures and by 30% in preformed biofilms. For WBPs, the percentages were, respectively, 52 and 48% (Figures 4B,D). In summary, we found that the Clx and Ch combination has significant antimicrobial activity and improves the treatment with Clx alone. This combination could enable a significant reduction in the antibiotic concentrations required to kill bacteria in different lifestyles, regardless of the biofilm biomass intensity and the antibiotic resistance pattern in NAS isolates.

**Table 3:** Effect of combined chitosan and cloxacillin on SBP and WBP NAS isolates in planktonic cultures and preformed biofilms.

Isolates	P-MBC[μg/ml]		B-MBC[μg/ml]	
	Clox. + Ch.	Clox./Clox. + Ch.	Clox. + Ch.	Clox./Clox. + Ch.
A. Strong biofilm producer NAS				
SBP1	0.0625	16	64	16
SBP2	0.0625	32	64	16
SBP3	0.125	16	64	16
SBP4	0.0625	32	64	16
SBP5	0.0625	16	64	16
SBP6	0.125	16	64	16
SBP7	0.125	32	64	8
SBP8	0.0625	16	64	16
SBP9	0.0625	16	64	4
SBP10	0.125	8	64	4
B. Weak biofilm producer NAS				
Planktonic cultures			Preformed biofilms	
WBP1	0.0625	32	4	8
WBP2	0.0625	16	2	4
WBP3	0.0625	32	4	8
WBP4	0.0625	32	4	8
WBP5	0.0625	32	4	4
WBP6	0.0625	16	2	4
WBP7	0.0625	32	4	8
WBP8	0.125	16	4	4
WBP9	0.0625	32	4	8
WBP10	0.125	16	4	4

A. Strong biofilms producer bacteria. B. Weak biofilms producer bacteria. Minimum bactericidal concentration of Clx + Ch in planktonic cultures (P-MBC), and in preformed biofilms (B-MBC). Bactericidal concentration ratio between Clx alone and Clx + Ch. Weak biofilm producer NAS.

## 4. Discussion

Until effective immunoprevention strategies are made available for this pathology, antibiotics will remain the main stay of treatment. Nevertheless, their progressive loss of effectiveness due to the speed at which bacteria develop virulence factors is becoming cause for global public health concern (WHO/FAO/OIE, 2016; Aidara-Kane et al., 2018). The ingrained belief that antimicrobials improve the overall health of live stock and lead to higher yields and better quality products has triggered their overuse (Gross, 2013; Michael et al., 2014). According to official FDA and EU reports, more than the 75% of the antibiotics sold in the US and the European Union are for veterinary use (Food and Drug Administration (FDA), 2010, 2018). In 2018 alone, more than 11,575 tons of antibiotics were administered in the US to food-producing animals; 42% of them were destined for cattle (Van Boeckel et al., 2015 Stevens et al., 2016) Food and Drug Administration (FDA), 2018.

NAS are one of the most prevalent pathogens of bovine IMIs. Although not always related to the severity of mastitis, they are usually associated with the persistence of infection and the worst antibiotic cure rates (Simojoki et al., 2012; Fry et al., 2014; Condas et al., 2017). Frequently, antibiotic therapy failures and persistent infections are not cannot be attributed to the presence of resistance genes in pathogens, which suggests that other mechanisms may play a relevant role in antibiotic cure rates (Lebeaux et al., 2014; Koo et al., 2017; Breser et al., 2018). Biofilm structures have been shown to act as protective shields against antibiotics (Hathroubi et al., 2017; Pedersen et al., 2021), and thus to favor the establishment of persistent and recurrent infections (Bjarnsholt et al., 2013; Flemming et al., 2016). NAS are more frequently capable than other mastitis-causing pathogens of producing biofilm biomass, which might therefore explain their high resistance rates (Tremblay et al., 2013; de Oliveira et al., 2016; Felipe et al., 2017; Srednik et al., 2017). Still, the influence of biofilm growth on antibiotic failure and infection chronicity and recurrence can be largely unpredictable (Flemming et al., 2016; Pedersen et al., 2021). In a murine IMI model, glands infected with a strong biofilm-forming *S. aureus* strain developed greater tissue inflammation, neutrophil recruitment, and functionality loss than those infected with a weak biofilm-forming strain (Gogoi-Tiwari et al., 2017). We found that NAS isolates from clinical BM, which were classified according to their biofilm biomass intensity, had significantly different antibiotic resistance patterns that were not species-dependent. A strong association was observed, in fact, between biofilm intensity and antibiotic resistance. Numerous other studies have determined that multiresistant bacteria isolated from different sources (burns, medical devices, chronic infections) are moderate or strong biofilm producers (Karigoudar et al., 2019; Mahmoudi et al., 2019; Folliero et al., 2021). This has not been observed for environmental bacteria of various origins (agricultural soils, surface water and sediments, plants, air, walls; Donadu et al., 2022; Sharan et al., 2023). Some authors contend that biofilm is synonymous with antibiotic resistance, because of its proficiency at transferring resistance genes and its innate phenotypic tolerance to antibiotics (Bowler et al., 2020; Trubenová et al., 2022). For this reason, new disease control strategies should focus on counteracting the protection offered by bacterial biofilms, since this could decrease resistance rates to the antimicrobials that are already in use. In this work, we found that the antimicrobial activity of the antibiotic Clx against the isolates was overall enhanced when it was combined with Ch, regardless of the bacterial lifestyle, the biofilm biomass intensity or the antibiotic resistance pattern. The data obtained suggest that the degree of protection conferred by biofilm against antibiotic treatment may depend on the intensity of its biomass, and that Ch could be useful to counteract this protection.

The antimicrobial concentrations which are effective for bacteria growing within biofilms have been reported to be significantly higher than for bacteria in planktonic cultures (Tremblay et al., 2014; Claessens et al., 2015; Breser et al., 2018). To gain a deeper understanding of the effect of biofilms on antimicrobial resistance and therapy failure, comparative and standardized antimicrobial assays on preformed biofilms are needed. Thieme et al. (2019) reviewed different reports that explored antimicrobial effects on preformed biofilms, and noted that the results of the assays could be interpreted in many ways. Different parameters like minimum biofilm eradication concentration (MBEC) or bactericidal biofilm concentration (BBC) do not always represent the antimicrobial concentrations required to kill bacteria. In some cases, 3 Log10 bacterial reductions were described with respect to control conditions or untreated bacteria (Tremblay et al., 2013; Brady et al., 2017; Cruz et al., 2018; Thieme et al., 2019).This means that the findings of different studies might not render comparable conclusions about the influence of biofilms on therapy efficacy (Thieme et al., 2019).

However, a few studies have focused on the antibiotic concentrations required to kill bacteria in terms of the biomass of their biofilms, and in comparison with their planktonic form. We found that the Clx concentrations needed to kill strong and weak biofilm producers (SBPs and WBPs) were only similar when the bacteria were grown planktonically. In preformed biofilms, much higher concentrations were needed to kill SBPs than WBPs. The addition of Ch to the Clx treatment not only reduced the concentration of the antibiotic needed to kill bacteria in the planktonic cultures but also in the preformed biofilms, independently of the biofilm intensity and the antibiotic resistance pattern. The antimicrobial effects of Ch and its mode of action have been explored in many microorganisms including viruses, bacteria, and fungi (Muxika et al., 2017; Sahariah and Másson, 2017; Verlee et al., 2017; Matica et al., 2019; Ke et al., 2021). Its versatility makes it an ideal candidate for combination with other compounds (Matica et al., 2019; Meng et al., 2021) and for the design of micro/nano-structures (Divya et al., 2017; Sahariah and Másson, 2017; Kravanja et al., 2019; Orellano et al., 2019), and there is wide evidence of its ability to inhibit biofilm biomass and eradicate preformed biofilms (Felipe et al., 2019; Orellano et al., 2019; Khan et al., 2020). For these reasons, it has been proposed (alone or combined with different antibiotics) as an alternative to improve the cure rates of infections caused by bacterial biofilms or multiresistant bacteria (Asli et al., 2017; Breser et al., 2018; Zhang et al., 2019; Meng et al., 2021). Indeed, Asli et al. (2017) used Ch by itself or combined with tilmicosin to treat a mammary gland infected by *S. aureus* in a murine model. Both the polymer on its own and its association with the antibiotic significantly reduced bacterial colonization, and the combined treatment was significantly better at decreasing the bacterial load inside the tissue than tilmicosin by itself.

Even though further research is essential to fully comprehend the extent of the influence exerted by bacterial biofilms on antimicrobial therapies, the findings available so far suggest that combining Ch and antibiotics could be a promising alternative to minimize antibiotic concentrations while improving the cure rates of bovine IMIs (Asli et al., 2017; Breser et al., 2018). In any case, novel treatment strategies will have to contemplate the central role played by biofilm in the development and persistence of bacterial infections.

## 5. Conclusion

According to the results of this study, strong and weak biofilm-producing NAS isolates from bovine mastitis have significantly different antibiotic resistance patterns. Such patterns are not species-dependent, but rather associated to the intensity of the biofilm biomass. The minimum bactericidal concentration of cloxacillin (Clx) was similar for both groups (strong and weak producers) when they were growing planktonically. A significantly higher concentration was required to kill bacteria in preformed biofilms, and in turn, this concentration was much higher for strong producers than for weak producers. These data confirm that the antibiotic concentration needed to kill bacteria in preformed biofilms is closely related to biofilm-forming ability. On the other hand, the addition of chitosan (Ch) to the Clx treatment made it possible to significantly reduce the bactericidal concentration of the antibiotic required for the two different lifestyles, regardless of the intensity of the biofilm biomass or the antibiotic resistance pattern of each isolate. These findings shed light on the influence exerted by bacterial biofilms on antibiotic treatments and antimicrobial resistance. Moreover, they provide evidence in favor of a therapeutic strategy that can mitigate such influence, and which could therefore be explored further as an alternative treatment for intramammary infections.

## Data availability statement

The raw data supporting the conclusions of this article will be made available by the authors, without undue reservation.

## Author contributions

MB and LT designed and carried out the experiments and interpreted the data. IB, LB, and PI discussed the experiments and results. MB, IB, and CP participated in design and discussion, and supervised the final draft of the manuscript. All authors contributed to the article and approved the submitted version.

## Funding

This work was supported by grants from the Argentinian Agency for the Promotion of Science and Technology (ANPCyT) (PICT 2016-1024 and PICT 2017-0822) and the National University of Villa Maria.

## Conflict of interest

The authors declare that the research was conducted in the absence of any commercial or financial relationships that could be construed as potential conflicts of interest.

## Publisher’s note

All claims expressed in this article are solely those of the authors and do not necessarily represent those of their affiliated organizations, or those of the publisher, the editors and the reviewers. Any product that may be evaluated in this article, or claim that may be made by its manufacturer, is not guaranteed or endorsed by the publisher.
