# Supplementary material for: Chitosan can improve antimicrobial treatment independently of bacterial lifestyle, biofilm biomass intensity and antibiotic resistance pattern in non-aureus staphylococci (NAS) isolated from bovine clinical mastitis
Source: Front Microbiol. 2023 Apr 21;14:1167693. doi: 10.3389/fmicb.2023.1167693 (PMC10162019; doi:10.3389/fmicb.2023.1167693)
Supplement: Supplementary file 1 [file Data_Sheet_1.PDF]

Supplementary Material

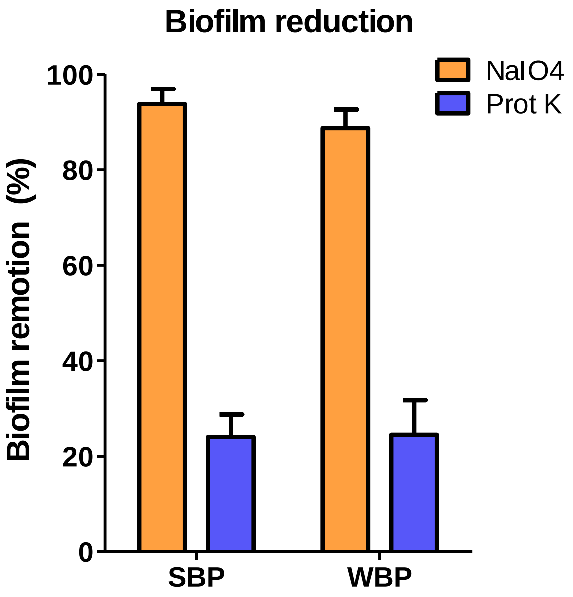

Supplementary Figure. Bacterial biomass reduction of SBP and WBP biofilms after treatment with metaperiodate or Proteinase K. Each NAS isolate from the SBP and WBP groups was grown on a flat bottom polystyrene plate with TSB for 24 h. The suspended bacteria were removed by washing, and the preformed biofilms were treated with 200uL of a NaCl 0.9% saline solution (control); 200 uL of sodium meta-periodate ($NaIO_4$) [40mM] to degrade 1,6-linked polysaccharides; or 200 uL of Proteinase K [0.1 mg/mL] to degrade proteins, for 2 h at 37ºC. The remaining biofilms were stained with crystal violet.Absorbance was measured at 590 nm using a Multiskan GO microplate spectrophotometer reader (TermoFisher Scientifc) and expressed as OD values. The bar graph shows the percentage of preformed biomass reduction after treatment with $NaIO_4$ or Proteinase K,with respect to the control. These experiments were performed three independent times with four biological replicates of each of the ten SBP and WBP isolates. Data were analyzed with one-way ANOVA followed by Bonferroni post-hoc, and are shown as mean ± SEM.
